# Eltrombopag directly activates BAK and induces apoptosis

**DOI:** 10.1038/s41419-023-05918-6

**Published:** 2023-07-01

**Authors:** Meng Chen, Lei Hu, Xuyuan Bao, Kaiqin Ye, Yunjian Li, Zhiyong Zhang, Scott H. Kaufmann, Jun Xiao, Haiming Dai

**Affiliations:** 1 Anhui Province Key Laboratory of Medical Physics and Technology, Institute of Health and Medical Technology, Hefei Institutes of Physical Science, Chinese Academy of Sciences, Hefei, 230031 China; 2 University of Science and Technology of China, Hefei, 230026 China; 3 Hefei Cancer Hospital, Chinese Academy of Sciences, Hefei, 230031 China; 4 School of Preclinical Medicine, Wannan Medical College, Wuhu, 241002 China; 5 Department of Physics, University of Science and Technology of China, Hefei, 230026 China; 6 Division of Oncology Research, Mayo Clinic, Rochester, MN 55905 USA; 7 Department of Molecular Pharmacology and Experimental Therapeutics, Mayo Clinic, Rochester, MN 55905 USA; 8 Department of Urology, The First Affiliated Hospital of USTC, Division of Life Sciences and Medicine, University of Science and Technology of China, Hefei, 230001 China

**Keywords:** Apoptosis, Solution-state NMR

## Abstract

Small molecule direct BAK activators can potentially be used for the development of anti-cancer drugs or as tools to study BAK activation. The thrombopoietin receptor agonist eltrombopag (Eltro) inhibits BAX activation and BAX-mediated apoptosis. Here we report that, in contrast to its function as a BAX inhibitor, Eltro directly binds BAK but induces its activation in vitro. Moreover, Eltro induces or sensitizes BAK-dependent cell death in mouse embryonic fibroblasts (MEFs) and Jurkat cells. Chemical shift perturbation analysis by NMR indicates that Eltro binds to the BAK α4/α6/α7 groove to initiate BAK activation. Further molecular docking by HADDOCK suggests that several BAK residues, including R156, F157, and H164, play an important role in the interaction with Eltro. The introduction of an R156E mutation in the BAK α4/α6/α7 groove not only decreases Eltro binding and Eltro-induced BAK activation in vitro but also diminishes Eltro-induced apoptosis. Thus, our data suggest that Eltro directly induces BAK activation and BAK-dependent apoptosis, providing a starting point for the future development of more potent and selective direct BAK activators.

## Introduction

BCL2 family proteins control mitochondrial outer membrane permeabilization (MOMP), a key step in the intrinsic apoptotic pathway [1–9]. Two pro-apoptotic effectors of this protein family, BAK, and BAX, directly cause MOMP when activated, leading to cytochrome c release and a downstream caspase cascade. The other BCL2 family proteins regulate MOMP through protein-protein interactions: The anti-apoptotic BCL2 subfamily members, including BCL2, BCLX_L_, and MCL1, inhibit MOMP by neutralizing pro-apoptotic family members; and the pro-apoptotic BCL2-homology (BH)3-only subfamily members, such as BIM and tBID, activate BAK or BAX indirectly or directly [10–15]. By neutralizing the anti-apoptotic BCL2 proteins, BH3-only proteins indirectly activate BAK/BAX [10, 11]. In addition, by directly binding BAK and BAX, thereby initiating activating conformational changes, some of the BH3-only proteins also serve as direct activators of BAK/BAX [11–15].

In living cells, BAK and BAX are in inactive states as monomers or as heterodimers neutralized by anti-apoptotic BCL2 family members [16–18]. Stimuli that induce apoptosis through the mitochondrial pathway often increase the expression and/or activation of BH3-only proteins, which then activate BAK and/or BAX. In cancer cells, however, the activator BH3-only proteins are often silenced or mutated, resulting in decreased mitochondrial priming and resistance to multiple anti-cancer treatments [2, 8, 19–22]. As a result, there is substantial interest in small molecules that can directly activate BAK or BAX, as these molecules could potentially induce cancer cell apoptosis directly or sensitize cancer cells to other anti-cancer treatments if suitably targeted to neoplastic cells.

BAK is normally present on the mitochondrial outer membrane, whereas BAX resides in the cytosol and requires mitochondrial translocation to induce MOMP [23]. Several small molecules that can directly activate BAX have been identified, including BAM7, BIF-44, and BTSA1 [24–26]. In contrast, pharmacologic BAK activators are more limited, with only a single study describing BKA-073, a small molecule identified through computational screening and subsequently found to directly activate BAK to induce lung cancer cell apoptosis [27]. Identification of additional small molecule direct BAK activators is, therefore, of interest.

Eltrombopag (Eltro) is an FDA-approved thrombopoietin receptor agonist that promotes platelet production for the treatment of chronic immune thrombocytopenia [28, 29]. Eltro, which reaches plasma concentrations up to 15–20 µM at doses typically administered clinically [30], binds the thrombopoietin receptor and induces an activating conformational change, leading to downstream JAK2/STAT5 activation and increased platelet production. A recent study found that Eltro also binds BAX to inhibit its activation [31]. Because BAX and BAK have similar structures, we evaluated the ability of Eltro to bind BAK and modulate its function. In contrast to Eltro’s inhibitory impact on BAX, we found that Eltro directly activates BAK and induces BAK-dependent apoptosis.

## Results

### Eltro directly binds and activates BAK in vitro

**Fig. 1:**
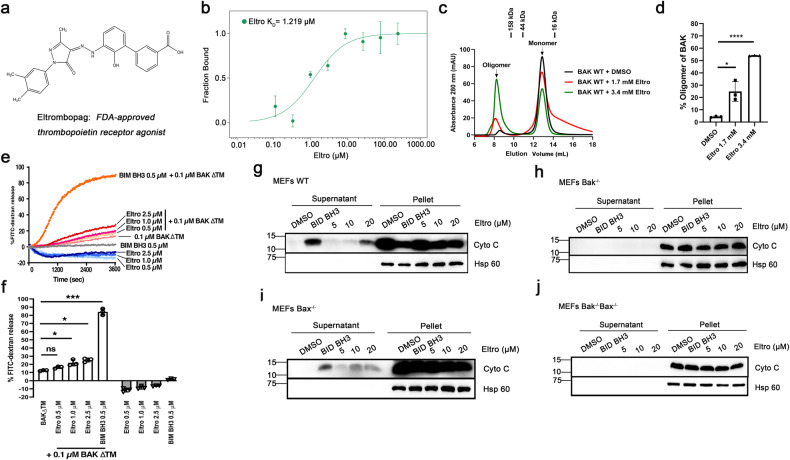
Eltrombopag directly binds BAK and induces BAK activation. **a** Structure of Eltrombopag (Eltro). **b** After recombinant BAKΔTM was labeled at cysteine using maleimide, microscale thermophoresis (MST) analysis was performed using a series of Eltro concentrations followed by plotting the fraction bound as a function of Eltro concentration. Inset, calculated dissociation constant based on MST assay. **c**, **d** After BAK and the indicated concentrations of Eltro or DMSO (negative control) were incubated in CHAPS buffer for 1 h at 25 °C, the mixture was applied to a Superdex75 size exclusion column on an FPLC, and absorbance at 280 nm was plotted as a function of elution volume. A representative experiment (**c**) and summary from three independent experiments (**d**) are shown. Labels at top of **c** indicate size markers. **e**, **f** After FITC-dextran-containing lisposome were incubated with the indicated concentration of BAK for 90 min at 25 °C, the percentage of FITC-dextran release was measured. A representative experiment (**e**) and summary from three independent experiments (**f**) are shown. **g**–**j** After mitochondria from WT (**g**), *Bak*^−/−^ (**h**), *Bax*^−/−^ (**i**), or *Bak*^−/−^*Bax*^−/−^ (**j**) MEFs were incubated with the indicated concentrations of Eltro or BID BH3 peptide (positive control) at 25 °C for 90 min, the supernatants and pellets were subjected to SDS-PAGE and immunoblotting. A representative result from three independent blotting experiments is shown in **g**–**j**.

To evaluate whether Eltro (Fig. 1a) binds BAK in vitro, we purified BAK lacking its transmembrane domain (BAKΔTM, Supplementary Fig. S1a, b) [32]. Microscale thermophoresis (MST) assay, which can use the concentration of ligand to induce fluorescence changes of a fluorescently labeled protein along microscopic temperature gradients to determine the affinity of a protein to a ligand, revealed that Eltro binds BAKΔTM directly, with an estimated dissociation constant (K_*D*_) of 1.2 ± 0.7 μM (Fig. 1b), raising the possibility that Eltro might be able to modulate BAK function.

To assess the impact of Eltro on BAK activation in vitro, we performed several assays [33]. First, the impact of Eltro in BAKΔTM oligomerization was examined by size exclusion chromatography. An average of 24% and 52% of BAKΔTM became oligomerized when BAKΔTM was incubated with 1.7 mM and 3.4 mM Eltro, respectively. Both the percentage of BAKΔTM oligomerized in 3.4 mM Eltro and the size of the oligomers are very similar to what was observed with the known BAK activator BID BH3 peptide (Fig. S1c). In contrast, BAKΔTM oligomers were almost undetectable in the absence of Eltro (Fig. 1c, d). Second, Eltro enhanced BAKΔTM-mediated liposome release in a concentration-dependent manner (Fig. 1e, f). Third, Eltro-induced cyto c release when incubated with mitochondria from wildtype (WT) MEFs (Fig. 1g). These results suggested that Eltro binds BAK and induces BAK activation in vitro.

Compared to BIM BH3 peptide-induced BAK-mediated liposome release, however, the Eltro-induced BAK-mediated liposome release was much lower (Fig. 1e). Moreover, less cyto c was released from mitochondria of WT MEFs after Eltro treatment than after BID BH3 peptide treatment (Fig. 1f and original blots for this figure and other figures in Supplementary Fig. S8). These observations suggested that Eltro is a weaker BAK activator than BIM BH3 or BID BH3 peptide.

### Eltro induces Bak-dependent apoptosis in MEFs

To evaluate whether Eltro-induced release of cyto c from mitochondria depends on Bak, we analyzed Eltro-induced cyto c release from mitochondria of *Bak*^−/−^, *Bax*^−/−^, and *Bak*^−/−^*Bax*^−/−^ (DKO) MEFs. This analysis showed that Eltro-induced cyto c release from mitochondria of *Bax*^*−/−*^ MEFs but not *Bak*^−/−^ and DKO MEFs (Fig. 1h–j), indicating a need for the presence of Bak.

**Fig. 2:**
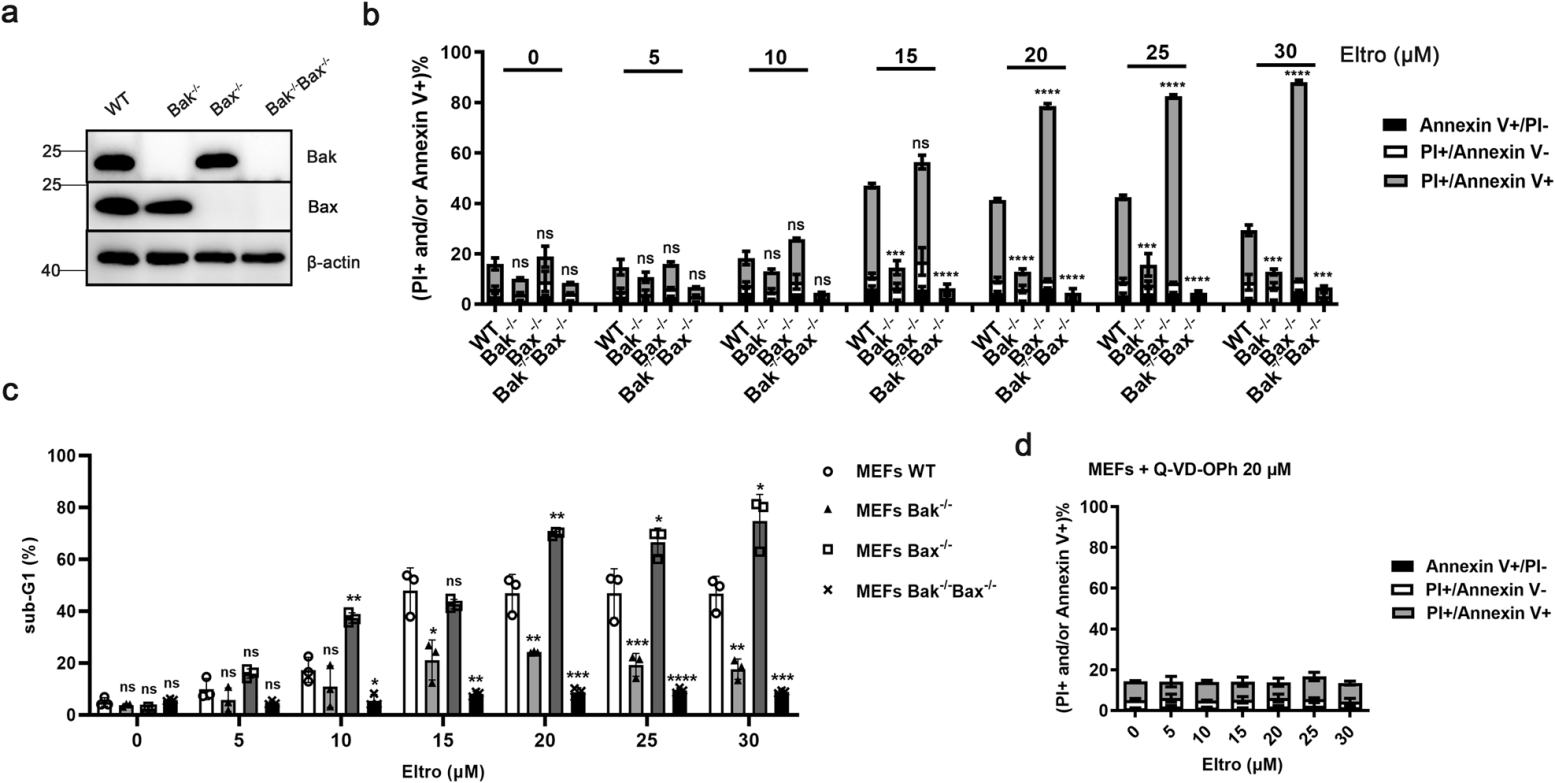
Eltrombopag induces BAK-dependent but not BAX-dependent apoptosis in MEFs. **a** Whole cell lysates from WT, *Bax*^−/−^*Bak*^−/−^, *Bak*^−/−^, or *Bax*^−/−^ MEFs were subjected to immunoblotting with the indicated antibodies. **b**, **c** After WT, *Bak*^−/−^, *Bax*^−/−^, or *Bax*^−/−^*Bak*^−/−^ MEFs were incubated with increasing Eltro concentrations (0–30 μM) for 24 h, the cells were subjected to Annexin V and PI analysis (**b**) or sub-G1 analysis (**c**) by flow cytometry. **d** After WT MEFs were incubated with increasing Eltro concentrations (0–30 μM) in the presence of Q-VD-OPh (20 μM) for 24 h, the cells were subjected to sub-G1 analysis by flow cytometry. Data in **b**–**d** represent mean ± SD from three independent experiments. Differences of Bak^−/−^, Bax^−/−^, or DKO compared to WT were determined by ANOVA. **b** The total of Annexin V+/PI−, PI+/Annexin V−, PI+/Annexin V+ cells were compared. *p < 0.05; **p < 0.01; ***p < 0.001; ****p < 0.0001; and ns, not significant.

To determine whether Eltro-induced Bak activation can trigger apoptosis, we treated WT, *Bak*^−/−^, *Bax*^−/−^, and *Bak*^−/−^*Bax*^−/−^ DKO MEFs (Fig. 2a) with Eltro and analyzed the induction of cell death using two different assays: An Annexin V/PI double staining assay and a flow cytometry-based assay for cells with fragmented DNA. Both of these assays showed that Eltro induces concentration-dependent apoptosis in WT MEFs, which is increased in *Bax*^−/−^ cells (Fig. 2b, c and Supplementary Fig. S2a), possibly because Bax sequesters Eltro and thus has inhibitory effects on Eltro-induced Bak-mediated apoptosis [31]. However, in *Bak*^−/−^ and DKO MEFs, Eltro-induced apoptosis was markedly diminished, suggesting that Eltro-induced apoptosis depends on Bak (Fig. 2b, c). Moreover, the pan-caspase inhibitor Q-VD-OPh also abolished Eltro-induced cell death in WT MEFs (Fig. 2d and Supplementary Fig. S2b), providing further evidence that the cells are dying by apoptosis.

### Eltro sensitizes Jurkat cells to S63845-induced apoptosis in a BAK-dependent manner

**Fig. 3:**
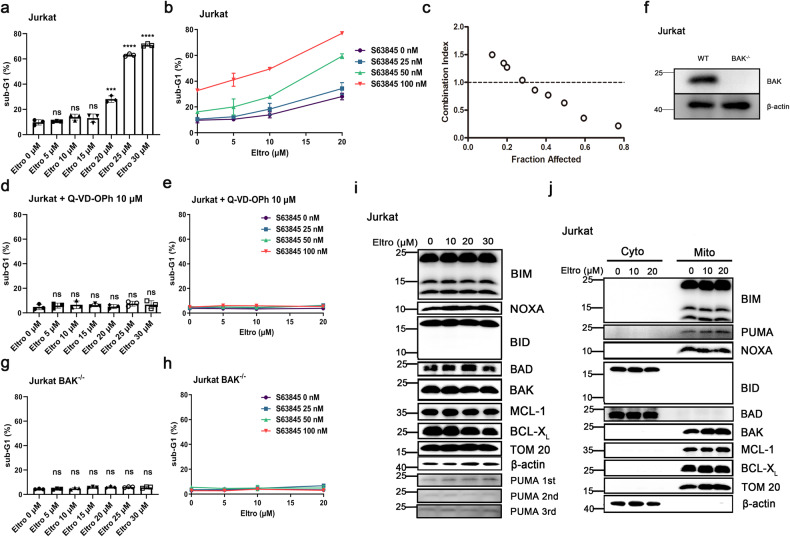
BAK-dependent Eltro/S63845 synergy in Jurkat cells. **a** After treatment with increasing Eltro concentrations (0–30 μM) for 24 h, Jurkat cells were subjected to sub-G1 analysis by flow cytometry. **b** After treatment with increasing concentrations of S63845 (0–100 nM) in combination with Eltro (0–20 μM) for 24 h, Jurkat cells were subjected to sub-G1 analysis by flow cytometry. **c** Combination index calculated using data from **b**. **d**, **e** Jurkat cells treated with Eltro alone (0–30 μM, **d**) or S63845 (0–100 nM) in combination with Eltro (0–20 μM) (**e**) in the presence of 10 μM Q-VD-OPh for 24 h were subjected to sub-G1 analysis by flow cytometry. **f** Whole cell lysates from WT and *BAK*^*−/−*^ Jurkat cells were subjected to immunoblotting with the indicated antibodies. Representative blotting data from three independent experiments are shown. **g**, **h** After treatment with Eltro alone (0–30 μM, **g**), or S63845 (0–100 nM) in combination with Eltro (0–20 μM) (**h**) for 24 h, *BAK*^*−/−*^ Jurkat cells were subjected to sub-G1 analysis by flow cytometry. **i** After Jurkat cells were treated with indicated concentrations of Eltro for 24 h in the presence of 10 μM Q-VD-OPh, whole cell lysates was subjected to immunoblotting for indicated antibodies. Representative blotting data from three independent experiments are shown. PUMA 1st, PUMA 2nd and PUMA 3rd are PUMA blots from three independent experiments. No reproducible change in PUMA expression was found. **j** After Jurkat cells were treated with the indicated concentrations of Eltro for 24 h in the presence of 10 μM Q-VD-OPh, cells were fractionated. The cytosol (Cyto) and mitochondrial fractions (Mito) were subjected to immunoblotting with the indicated antibodies. Data in **a**, **b**, **d**, **e**, **g**, **h** represent mean ± SD from three independent experiments.

To limit the influence of BAX-mediated apoptosis, we evaluated Eltro-induced apoptosis in Jurkat cells, which have an ~8-fold molar excess of BAK to BAX and are more dependent on BAK than BAX for responding to most apoptotic stimuli [32, 34]. Eltro monotherapy induced apoptosis in these cells, but only when its concentration was higher than 20 μM (Fig. 3a). Based on the hypothesis that high levels of anti-apoptotic proteins such as BCLX_L_ and MCL1 observed in Jurkat cells [32, 34] might be responsible for this limited sensitivity, we evaluated the ability of the selective MCL1 inhibitor S63845 [35] to sensitize the Jurkat cells to Eltro. These studies showed that S63845 concentrations as low as 50 nM could sensitize Jurkat cells to Eltro and that effects of the two agents were synergistic (Fig. 3b, c). Q-VD-OPh inhibited cell death induced by both Eltro monotherapy and the S63845/Eltro combination (Fig. 3d, e), confirming the requirement for caspase activity. Moreover, *BAK* knockout (Fig. 3f) abolished the induction of cell death by both Eltro monotherapy and the S63845/Eltro combination (Fig. 3g, h). To assess whether BH3-only proteins might have been upregulated or modified to cause BAK activation, we evaluated the expression of BCL2 family proteins upon Eltro treatment but did not find significant changes (Fig. 3i). PUMA showed slight increase from one experiment, but did not show reproducible change (Fig. 3i). Likewise, we did not find significant translocations of BH3-only proteins to mitochondria, suggesting that BAK activation was not induced by BH3-only proteins (Fig. 3j). Moreover, BID cleavage was not observed after Eltro treatment (Fig. 3i), ruling out the possibility of death receptor pathway activation in these type II (BID-dependent) cells. Taken together, these analyses suggested that Eltro directly activates BAK and induces BAK-dependent apoptosis in different cell lines.

### Eltrombopag binds the α4/α6/α7 groove of BAK to initiate activation

**Fig. 4:**
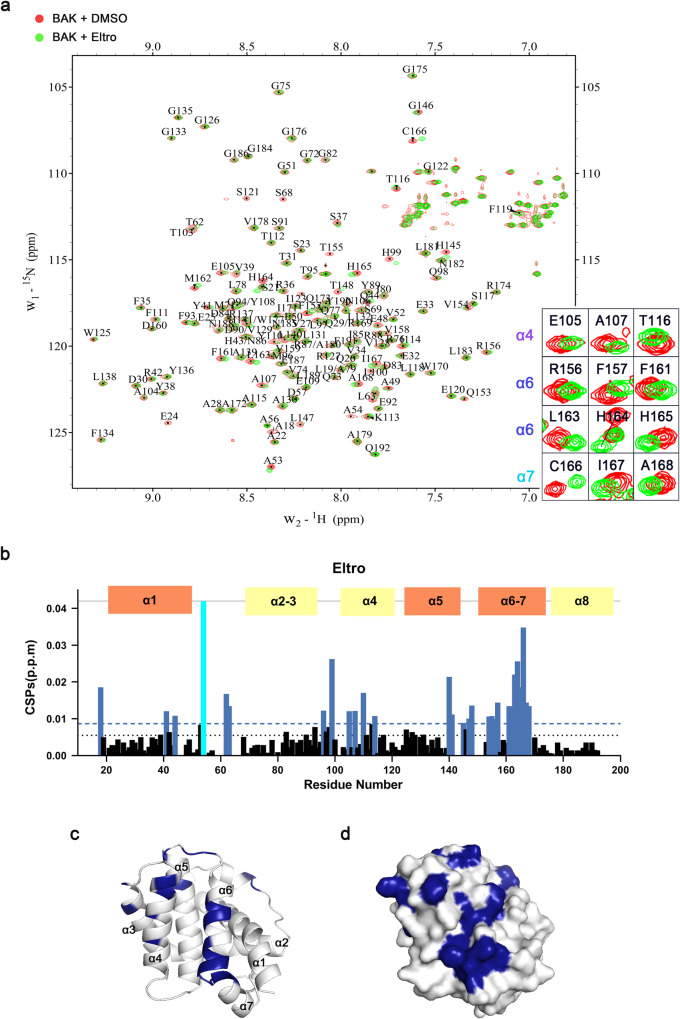
BAK 15–186 ^1^H,^15^N-HSQC perturbation indicates Eltrombopag binds to the α4/α6/α7 groove. **a** ^1^H,^15^N-HSQC spectra of 0.2 mM ^15^N-labeled BAK 15–186 were recorded in the presence of DMSO (control, red) or 4.0 mM Eltro (green) shown in different colors. Peaks on the right indicate the perturbations at selected residues. **b** Chemical shift perturbations (CSPs) were plotted as a function for BAK residues, when ^1^H,^15^N-HSQC spectra of BAK 15–186 were recorded after titration of Eltro at the ratio of protein:Eltro=1:20. CSPs per BAK residue were calculated using the formula CSPs = [(∆δNH^2^ + (∆δN/25)^2^)/2]^1/2^. The threshold value was defined as average CSP value plus 1/2 standard deviation. **c**, **d** Surface and cartoon mixed model (**c**) or surface model (**d**) of BAK (PDB:2IMS) showing residues (indicated in blue) that have obvious chemical shift changes or disappearance of signals (peak intensities decreased to the level of noise) in the ^1^H,^15^N-HSQC spectra after the perturbation by Eltro.

**Fig. 5:**
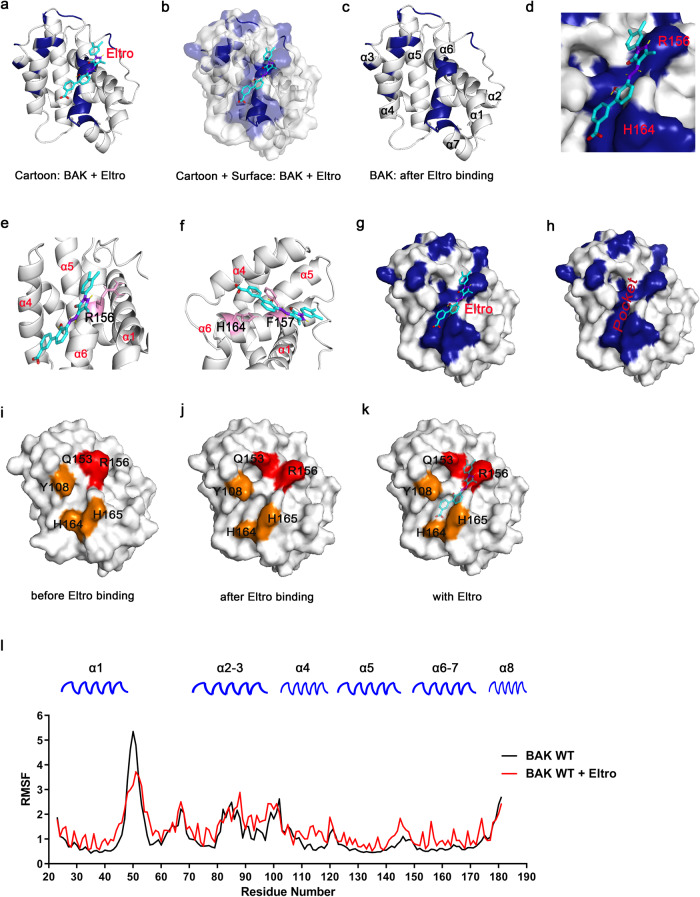
Binding of Eltrombopag to the BAK α4/α6/α7 groove induces a BAK conformational change. **a**–**c** Overall view of Eltro binding to BAK at the α4/α6/α7 groove as depicted in a cartoon model (**a**) and a cartoon plus surface model (**b**) without Eltro (**c**). **d** Closeup view of Eltro binding to the BAK α4/α6/α7 groove showing involvement of BAK R156 and H164 in Eltro binding. **e**, **f** Cartoon models showing molecular interactions of Etro with R156 (**e**), F157, and H164 **f**. **g**, **h** Surface models showing Eltro induces a hydrophobic pocket on BAK α4/α6/α7 groove. Models show BAK with Eltro (**g**) and without Eltro (**h**). **i**–**k** Conformational change of two groups of BAK residues (Group #1: Q153 and R156 shown in red; Group #2: Y108, H164, and H165 shown in orange) induced by Eltro to expose the α4/α6/α7 groove. **i** before adding Eltro; **j** after adding Eltro; **k**, with Eltro. **l** Comparison of RMSF per BAK residue when BAK structure (PDB: 2IMS) was used to perform molecular dynamics simulations with or without Eltro.

To provide further insight into the mechanism by which Eltro induces BAK activation, we performed an NMR titration assay to identify the Eltro binding site on BAK. Briefly, after ^15^N-labeled BAK 15–186 was purified and ^1^H,^15^N-HSQC signals were assigned as previously described (Fig. 4a) [36], chemical shift perturbations were measured upon the addition of Eltro. Several residues, including E105, A107, T116, R156, F157, F161, L163, H164, H165, C166, I167, and A168, exhibited focal chemical shift changes upon Eltro addition, while the residue A54 exhibited ligand-induced signal disappearance (Fig. 4a, b and Supplementary Fig. S3). When mapped onto the structure of BAK (PDB ID: 2IMS [37], Fig. 4c, d), most of these residues were located in the α4/α6/α7 groove previously implicated in binding the BH3 domain of the BH3-only protein BMF [36]. To further investigate how Eltro activates BAK, we performed molecular modeling using HADDOCK, an algorithm for predicting the structure of protein complexes from NMR chemical shift data [38]. A cluster of structures that had the best HADDOCK score and was most prevalent (230 out of 400 structures) was used for further analysis, while the other clusters all were less abundant (<40 structures each, Table 1). Analysis of the complex (Fig. 5a-c) suggested that Eltro bound to mostly the α6 side (i.e., R156 and H164) of the α4/α6/α7 groove (Fig. 5d). Several interactions between Eltro and amino acids comprising α6 were identified by this analysis. First, the long side chain of BAK R156 formed hydrophobic interactions with the benzoate ring of Eltro (Fig. 5e). Second, the imidazole ring of BAK H164 and benzene ring of F157 formed stacking interactions with the dimethylbenzene ring of Eltro (Fig. 5f). These interactions are all in agreement with the NMR chemical shift perturbation analysis.

**Table 1:** Statistics of Eltro docking to BAK (PDB: 2IMS [37]) by HADDOCK.

Cluster Number	HADDOCK score	Cluster size	RMSD^a^	Z-Score
1	−25.8 ± 1.3	230	0.8 ± 0.0	−2.4
6	−22.7 ± 0.9	9	0.8 ± 0.0	−0.7
5	−22.4 ± 1.0	15	0.8 ± 0.0	−0.6
7	−21.8 ± 2.6	9	0.9 ± 0.0	−0.2
4	−21.0 ± 3.6	21	0.8 ± 0.0	0.2
2	−20.6 ± 0.6	37	0.8 ± 0.0	0.4
8	−20.4 ± 2.2	9	0.8 ± 0.1	0.5
9	−20.3 ± 2.7	7	0.8 ± 0.0	0.6
3	−20.0 ± 1.9	25	0.9 ± 0.1	0.8
10	−18.7 ± 0.4	5	0.8 ± 0.0	1.4

^a^RMSD from the overall lowest-energy structure.

### Eltrombopag induces BAK a conformational change at α1 helix

Comparison of unliganded BAK and the predicted structure after Eltro binding suggested that the hydrophobic groove became wider and deeper to fit Eltro (Fig. 5g, h). This hydrophobic groove is formed by two groups of residues, Q153/R156 and Y108/H164/H165, which opened wider and deeper after Eltro binding (Fig. 5i–k).

BAK activation includes extensive conformational changes [39, 40]. To identify additional conformational changes that might be triggered by Eltro binding, we conducted comprehensive, microsecond, isobaric-isothermal MD simulations of BAK bound to Eltro. This approach suggested that part of BAK α1 (residues 48–52) underwent an extensive conformational change after Eltro bound (Fig. 5l). While previous studies have suggested that BAK α1 conformational change is essential for BAK activation [40, 41], our data raise the possibility that the α1 conformational change after Eltro binding might facilitate the interactions of BAK with mitochondrial lipids that play a role in its activation [42].

### Mutation in the α4/α6/α7 groove inhibits Eltro-induced BAK activation and apoptosis

**Fig. 6:**
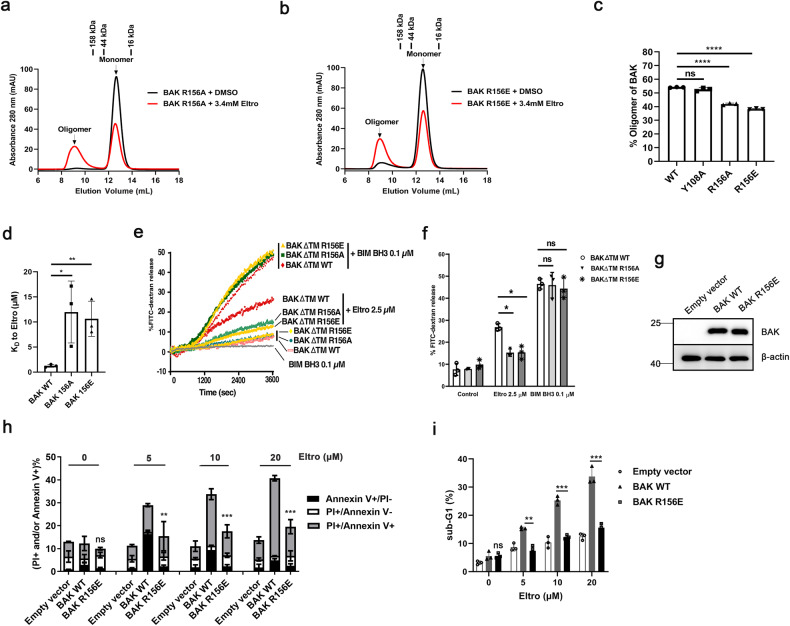
Dependence of Eltrombopag binding and subsequent apoptosis on BAK α4/α6/α7 groove. **a**, **b** After BAKΔTM R156A (**a**) or R156E (**b**) and indicated concentrations of Eltro were incubated in CHAPS buffer for 1 h at 25 °C, the mixtures were separated by FPLC on a Superdex75 size exclusion column and absorbance at 280 nm was plotted as a function of elution volume. Labels on top indicate size markers. **c** After BAKΔTM WT and different BAKΔTM mutants were analyzed by the BAK oligomerization assay shown in **a**, **b**, the percentage of BAK oligomers were calculated. **d** After recombinant BAKΔTM WT, BAKΔTM R156A or R156E was labeled at cysteine using maleimide, the dissociation constants (*K*_D_) was evaluated by MST analysis using increasing concentrations of Eltro. A summary of data from three independent experiments is shown. **e**, **f**. Liposome permeabilization assay was performed in the presence of 50 nM BAKΔTM WT, BAKΔTM R156A, or BAKΔTM R156E and the indicated concentrations of Eltro or BIM BH3 peptide as a positive control. A representative experiment (**e**) and a summary from three independent experiments (**f**) are shown. **g** *Bak*^−/−^*Bax*^−/−^ MEFs were transduced with WT BAK or BAK R156E. After 2 weeks of selection, a pool of cells was subjected to western blot. **h**, **i** *Bak*^−/−^*Bax*^−/−^ MEFs reconstituted with WT BAK or BAK R156E were treated with the indicated Eltro concentrations for 24 h and analyzed by Annexin V/PI staining (**h**) or sub-G1 analysis (**i**). **c**, **d**, **f**, **h**, **i** Differences between BAK mutants and WT were assessed by ANOVA. *p < 0.05; **p < 0.01; ***p < 0.001; ****p < 0.0001; and ns, not significant.

To validate the predicted Eltro binding site on BAK, we introduced a series of mutations into BAK (Supplementary Fig. S1a, b), and evaluated BAK activation by size exclusion chromatography. We found that BAK R156A and R156E mutations in the α4/α6/α7 groove, which affect binding to the benzoate moiety, inhibited Eltro-induced but not BID BH3-induced BAKΔTM oligomerization, whereas the Y108A mutation did not inhibit Eltro-induced BAKΔTM oligomerization (Fig. 6a–c, Supplementary Figs. S4a–c, S5a). Several other mutations of the α4/α6/α7 groove (F157A, D160A, and H165A) prompted oligomerization of BAKΔTM without Eltro and, therefore, were not used for further studies (Supplementary Fig. S5b–d). Nonetheless, the effects of the BAK R156A and R156E mutations provided further support for the possibility that Eltro binds to the α4/α6/α7 groove and provided the starting point for additional studies.

MST assays indicated the R156A and R156E BAK mutants both exhibited significantly reduced binding to Eltro (Fig. 6d, Supplementary Fig. S6). Moreover, Eltro-induced BAK-mediated liposome release was also significantly inhibited by R156A and R156E mutations, while liposome release induced by the BIM BH3 peptide, which binds the BAK α3/α4/α5 groove, was not affected by either mutation (Fig. 6e, f). In further experiments, we reconstituted DKO MEFs with either human BAK WT or R156E (Fig. 6g). WT BAK restored Eltro-induced apoptosis in the DKO MEFs, whereas BAK R156E, which exhibits reduced Eltro binding, resulted in much lower levels of Elro-induced apoptosis (Fig. 6h, i and Supplementary Fig. S7). Taken together, these data suggest that the interaction Eltro with BAK cells plays a critical role in Eltro-induced apoptosis in intact cells.

## Discussion

In this study, we have demonstrated that Eltro directly binds to and activates BAK in vitro. In addition, Eltro induces BAK-dependent apoptosis in MEFs and synergizes with BH3 mimetics in Jurkat cells. Further experiments have suggested that Eltro binds the α4/α6/α7 groove of BAK and the introduction of a mutation in this groove inhibits Eltro/BAK binding and Eltro-induced apoptosis. Our study not only provides a start point for deriving more efficient small molecule BAK activators, but also provides a possible tool for further study of BAK activation.

Mutations or diminished expression of BH3-only proteins have been reported to play an important role in tumorigenesis and drug resistance [19–22, 43–47]. Previous studies have indicated that BH3-only proteins induce apoptosis through two mechanisms: (1) Inhibition of anti-apoptotic BCL2 proteins to prevent their binding and sequestration of activated BAK or BAX; and (2) direct activation of BAK and/or BAX [10–15]. Several BH3-only proteins, including BIM, PUMA, tBID, NOXA, BMF, and HRK, have been reported to directly activate BAK and/or BAX [11–15, 36, 48–52]. When BH3-only proteins sustain loss-of-function mutations or are silenced, the related cell death pathway will not be activated, which can result in tumorigenesis and resistance to cancer treatments [19–22, 43–47]. To overcome the resistance that develops in this fashion, several small molecules that directly activate BAX have been developed [24–26]. In contrast, small molecules that directly activate BAK are still lacking. Accordingly, the identification of small molecules that can directly activate BAK might be useful for the induction of apoptosis in cancer cells, either directly or by sensitizing them to other anti-cancer drugs such as BH3 mimetics.

In contrast to its inhibitory effect on BAX activation [31], Eltro exhibits the ability to directly activate BAK in vitro as shown by several independent methods, including a BAK oligomerization assay, an assay for BAK-mediated liposome permeabilization, and an assay for cyto c release from mitochondria. Based on these observations, we evaluated the impact of Eltro on cell survival and found that Eltro induces Bak-dependent apoptosis in MEFs. Although Eltro does not induce Jurkat cell death at concentrations lower than 15 μM, a concentration of 5 μM Eltro sensitizes Jurkat cells to S63845. In addition, both Eltro- and S63845/Eltro-induced apoptosis are inhibited by *BAK* gene interruption in Jurkat cells. Interestingly, Eltro-induced more apoptosis in *Bax*^*−/−*^ MEFs than in WT cells, possibly because the Bax in WT MEFs sequesters Eltro so that less Eltro is available to activate Bak compared to Bax^−/−^ cells [31]. Nonetheless, the observation cells lacking Bak (either *Bak*^*−/−*^ or *Bax*^*−/−*^*Bak*^*−/−*^ DKO cells) fail to undergo apoptosis suggesting that BAK activation induced by Eltro causes cell death in these cells.

BAK activation has been suggested to be initiated at the α3/α4/α5 groove or the α4/α6/α7 groove [15, 33, 36, 52]. Through NMR titration, we found that Eltro induces chemical shifts mostly along the α4/α6/α7 groove. Our molecular docking study also suggested a model in which Eltro binds the α4/α6/α7 groove. Further experiments indicated that mutations in this groove, such as R156A or R156E, not only impaired Eltro/BAK interactions, but also inhibited Eltro-induced BAK-mediated liposome release. In contrast, neither mutation affected the ability of BID BH3 or BIM BH3 peptide, which bind to the α3/α4/α5 goove, to induce liposome release or BAK activation, further supporting the hypothesis that Eltro induces BAK activation through the α4/α6/α7 groove that we previous implicated in BMF BH3- or HRK BH3-induced BAK activation [36]. Although the NMR titration results indicate that residues involved in Eltro binding are somewhat different from those that bind BMF BH3 or HRK BH3, the binding groove is the same [36], again suggesting that activators binding on sites other than the canonical α3/α4/α5 groove have the ability to induce BAK conformational change and BAK activation.

By comparing the Eltro binding sites on BAX and BAK, we found some differences. First, Eltro binds BAX in the groove formed by α1/α6 [31], while Eltro binds BAK in the α4/α6/α7 groove. In both cases helix α6 of BAX or BAK is involved. Moreover, some residues at corresponding sites on the α6 are also involved in the binding to both proteins. For example, L163 on BAK (which corresponds residue R145 on BAX) is involved in Eltro binding. Despite Eltro binding to some of the corresponding residues in BAX and BAK, there are two potential explanations for the difference in outcomes of the binding. First, the different binding site between Eltro on BAX (the α1/α6 groove) and BAK (the α4/α6/α7 groove) might explain why Eltro binding inhibits BAX and activates BAK. Alternatively, these different outcomes might reflect a difference in the affinity of the binding. Eltro binds to BAX with a K_*D*_ of 0.145 µM [31], while it binds to BAK with a K_*D*_ of about 1.2 µM. Thus, it might be easier for Eltro to dissociate from BAK than from BAX after it induces BAX/BAK conformational changes.

Although our results suggest that Eltro induces BAK activation in vitro and in vivo, the present observations are only the starting point for the development of a potent and selective BAK activator. Study of Eltro derivatives is required to obtain a better direct BAK activator to induce cancer cell death because: (1) the efficacy of Eltro in activating BAK and inducing cell death is much lower than BIM BH3 or BID BH3; and (2) Eltro directly induces BAK activation but inhibits BAX activation, whereas it might be possible to develop a BAK activator without the BAX inhibition properties. If the study of Eltro derivatives yields a more efficacious and selective BAK activator, further studies will also likely be needed assure selective killing of cancer cells and not normal cells. While it has previously been reported that BAK levels are low in normal postnatal cells and higher in tumor cells [53], providing a potential opportunity for selective tumor cell killing using a BAK direct activator, it is possible that such an activator will be even more selective if administered as a payload attached to an antibody that selectively binds to certain tumor cells.

In summary, we found that Eltro directly binds and activates BAK in vitro. Further NMR titration and mutation analyses indicated that Eltro binds the α4/α6/α7 groove of BAK. Depletion of BAK or certain mutations of the Eltro binding site on BAK inhibits Eltro-induced apoptosis, demonstrating the importance of Eltro/BAK binding for BAK-mediated apoptosis.

## Materials and methods

### Preparation of FITC-dextran lipid vesicles

1-Palmitoyl-2-oleoyl-*sn*-glycero-3-phosphocholine, 1-plamitoyl-2-oleoyl-*sn*-glycero-3- phosphoethanolamine, l-α-phosphatidylinositol, cardiolipin, cholesterol, and 18:1 DGS-nitrilotriacetic acid (Ni^2+^) at a weight ratio of 39:22:9:20:8:2 were dried as thin films in glass test tubes under nitrogen and then under vacuum for 16 h. A lower concentration (2%) of DGS-Ni^2+^ was used to reduce the baseline of liposome permeabilization [54]. To encapsulate FITC-labeled dextran 10 (F-d10), 50 mg of lipid in 1 ml of 20 mm HEPES, 150 KCl (pH 7.0) was mixed with 50 mg of F-d10, sonicated, and extruded 15 times through a 100-nm polycarbonate membrane. Untrapped F-d10 was removed by gel filtration on Sephacryl S-300 HR (GE Healthcare). Phosphate was determined by colorimetric assay (Abcam, Cambridge, UK).

### Liposome release assay (modified from Oh et al. [55])

Release of F-d10 from large unilamellar vesicles was monitored by fluorescence dequenching using a fluorimetric plate reader. After purified WT or mutant His_6_-BakΔTM with Eltro was added to large unilamellar vesicles (final lipid concentration 10 μg/ml), 96-well plates were incubated at 25 °C with mixing and assayed (excitation 485 nm, emission 538 nm) every 10 s. F-d10 release was quantified by the equation ((*F*_sample_ − *F*_blank_)/(*F*_Triton_ − *F*_blank_) × 100%), where *F*_sample_, *F*_blank_, and *F*_Triton_ are fluorescence of reagent-, buffer-, and 1% Triton-treated large unilamellar vesicles.

### Apoptosis assays

To assay for sub-G1 DNA content, cells were collected after treatments, stained with propidium iodide in 0.1% (w/v) sodium citrate containing 0.1% (w/v) Triton X-100, and analyzed by flow cytometry [56]. Alternatively, cells were stained with APC-labeled Annexin V and PI, subjected to flow cytometry on a Beckman CytoFLEX, and analyzed using Beckman CytoExpert software.

### Molecular docking

Haddock2.4 online software was used for protein-ligand docking [38, 57]. NMR amino acid offset sites were used as active residues, and docking was carried out according to the optimized parameters for protein-ligand docking. From the top ten clusters with the best docking results, the number 1 best structure with the highest absolute value of Z-Score and the largest Cluster size was selected as the reliable docking result for further analysis.

### All-atom MD simulations

Using the model derived from HADDOCK, an independent MD simulation was conducted using the Amber20 software package (San Francisco, CA, USA) [58] and the Amber ff99SB force filed. The periodic boundary condition with a truncated octahedral box was used. The minimum distance between the solute and the box boundary was 10 Å. The box was filled with OPC water molecules and ions [59], reaching a salt concentration of 150 mM NaCl. To remove bad contacts, the waters, and ions were initially minimized for 1000 steps using the steepest decent method for the first 500 steps and then the conjugate gradient for the last 500 steps, with the position of protein fixed (force constant was 100 kcal mol^−1^ Å^−2^). After that, a heat-up MD was run at a constant volume. The system was heated from 100 K to 300 K for 100 ps with a weak restraint of 100 kcal mol^−1^ Å^−2^ on the protein. A free MD simulation of 1 μs in which the restraints on the protein were then removed under the NPT condition utilizing the GPU accelerated pmemd.cuda code. Temperature was regulated using the Langevin dynamics with a collision frequency of 1.0 ps^−1^ [60]. Pressure was controlled with isotropic position scaling at 1 bar with a relaxation time of 2.0 ps. All the bonds involving hydrogen atoms were constrained using the SHAKE algorithm [61]. A 2 fs integration step was used. The long-range electrostatic interaction was calculated using the PME method with a 9 Å cutoff for the range-limited non-bonded interaction [62]. Materials and some other Methods are described in the Supplementary Information.

## Supplementary information

Supplemental material

aj-checklist

## Data Availability

This study includes no data deposited in external repositories. All the raw data reported in this paper will be shared by the lead contact upon request.

## References

[1] Strasser A, Vaux DL (2020). Cell death in the origin and treatment of cancer. Mol Cell.

[2] Singh R, Letai A, Sarosiek K (2019). Regulation of apoptosis in health and disease: the balancing act of BCL-2 family proteins. Nat Rev Mol Cell Biol.

[3] Kale J, Osterlund EJ, Andrews DW (2018). BCL-2 family proteins: changing partners in the dance towards death. Cell Death Differ.

[4] Diepstraten ST, Anderson MA, Czabotar PE, Lessene G, Strasser A, Kelly GL (2022). The manipulation of apoptosis for cancer therapy using BH3-mimetic drugs. Nat Rev Cancer.

[5] Czabotar PE, Lessene G, Strasser A, Adams JM (2014). Control of apoptosis by the BCL-2 protein family: implications for physiology and therapy. Nat Rev Mol Cell Biol.

[6] Dellbridge AR, Grabow S, Strasser A, Vaux DL (2016). Thirty years of BCL-2: translating cell death discoveries into novel cancer therapies. Nat Rev Cancer.

[7] Moldoveanu T, Follis AV, Kriwacki RW, Green DR (2014). Many players in BCL-2 family affairs. Trends Biochem Sci.

[8] Bhola PD, Letai A (2016). Mitochondria-judges and executioners of cell death sentences. Mol Cell.

[9] Roberts AW, Wei AH, Huang DCS (2021). BCL2 and MCL1 inhibitors for hematologic malignancies. Blood..

[10] Willis SN, Fletcher JI, Kaufmann T, van Delft MF, Chen L, Czabotar PE (2007). Apoptosis initiated when BH3 ligands engage multiple Bcl-2 homologs, not Bax or Bak. Science..

[11] Llambi F, Moldoveanu T, Tait SW, Bouchier-Hayes L, Temirov J, McCormick LL (2011). A unified model of mammalian BCL-2 protein family interactions at the mitochondria. Mol Cell.

[12] Leshchiner ES, Braun CR, Bird GH, Walensky LD (2013). Direct activation of full-length proapoptotic BAK. Proc Natl Acad Sci USA.

[13] Kuwana T, Bouchier-Hayes L, Chipuk JE, Bonzon C, Sullivan BA (2005). BH3 domains of BH3-only proteins differentially regulate Bax-mediated mitochondrial membrane permeabilization both directly and indirectly. Mol Cell..

[14] Gavathiotis E, Suzuki M, Davis ML, Pitter K, Bird GH, Katz SG (2009). BAX activation is initiated at a novel interaction site. Nature..

[15] Dai H, Smith A, Meng XW, Schneider PA, Pang YP, Kaufmann SH (2011). Transient binding of an activator BH3 domain to the Bak BH3-binding groove initiates Bak oligomerization. J Cell Biol.

[16] Dai H, Ding H, Meng XW, Peterson KL, Schneider PA, Karp JE (2015). Constitutive BAK activation as a determinant of drug sensitivity in malignant lymphohematopoietic cells. Genes Dev.

[17] O’Neill KL, Huang K, Zhang J, Chen Y, Luo X (2016). Inactivation of prosurvival Bcl-2 proteins activates Bax/Bak through the outer mitochondrial membrane. Genes Dev.

[18] Peña-Blanco A, García-Sáez AJ (2018). Bax, Bak and beyond – mitochondrial performance in apoptosis. FEBS J.

[19] Beroukhim R, Mermel CH, Porter D, Wei G, Raychaudhuri S, Donovan J (2010). The landscape of somatic copy-number alteration across human cancers. Nature..

[20] Rampino N, Yamamoto H, Ionov Y, Li Y, Sawai H, Reed JC (1997). Somatic frameshift mutations in the BAX gene in colon cancers of the microsatellite mutator phenotype. Science..

[21] Certo M, Del Gaizo Moore V, Nishino M, Wei G, Korsmeyer S (2006). Mitochondria primed by death signals determine cellular addiction to antiapoptotic BCL-2 family members. Cancer Cell.

[22] Vo TT, Letai A (2010). BH3-only proteins and their effects on cancer. Adv Exp Med Biol.

[23] Goping IS, Gross A, Lavoie JN, Nguyen M, Jemmerson R, Roth K (1998). Regulated targeting of BAX to mitochondria. J Cell Biol.

[24] Gavathiotis E, Reyna DE, Bellairs JA, Leshchiner ES, Walensky LD (2012). Direct and selective small-molecule activation of proapoptotic BAX. Nat Chem Biol.

[25] Pritz JR, Wachter F, Lee S, Luccarelli J, Wales TE, Cohen DT (2017). Allosteric sensitization of proapoptotic BAX. Nat Chem Biol.

[26] Reyna DE, Garner TP, Lopez A, Kopp F, Choudhary GS, Sridharan A (2017). Direct activation of BAX by BTSA1 overcomes apoptosis resistance in acute myeloid leukemia. Cancer Cell.

[27] Park D, Anisuzzaman ASM, Magis AT, Chen G, Xie M, Zhang G (2012). Discovery of small molecule Bak activator for lung cancer therapy. Theranostics..

[28] Corman SL, Mohammad RA (2010). Eltrombopag: a novel oral thrombopoietin receptor agonist. Ann Pharmacother.

[29] Ghanima W, Cooper N, Rodeghiero F, Godeau B, Bussel JB (2019). Thrombopoietin receptor agonist: ten years later. Haematologica..

[30] Lam MS (2010). Review article: second-generation thrombopoietin agents for treatment of chronic idiopathic thrombocytopenic purpura in adults. J Oncol Pharm Pract.

[31] Spitz AZ, Zacharioudakis E, Reyna DE, Garner TP, Gavathiotis E (2021). Eltrombopag directly inhibits BAX and prevents cell death. Nat Commun.

[32] Dai H, Meng WX, Lee SH, Schneider PA, Kaufmann SH (2009). Context-dependent Bcl-2/Bak interactions regulate lymphoid cell apoptosis. J Biol Chem.

[33] Dai H, Pang YP, Ramirez-Alvarado M, Kaufmann SH (2014). Evaluation of the BH3-only protein Puma as a direct Bak activator. J Biol Chem.

[34] Dai H, Ding H, Peterson KL, Meng XW, Schneider PA, Knorr KLB (2018). Measurement of BH3-only protein tolerance. Cell Death Diff.

[35] Kotschy A, Szlavik Z, Murray J, Davidson J, Maragno AL, Toumelin-Braizat GL (2016). The MCL1 inhibitor S63845 is tolerable and effective in diverse cancer models. Nature..

[36] Ye K, Meng WX, Sun H, Wu B, Chen M, Pang Y-P (2020). Characterization of an alternative BAK-binding site for BH3 peptides. Nat Commun.

[37] Moldoveanu T, Liu Q, Tocilj A, Watson M, Shore G, Gehring K (2006). The X-ray structure of a BAK homodimer reveals an inhibitory zinc binding site. Mol Cell.

[38] Van Zundert GCP, Rodrigues JPGLM, Trellet M, Schmitz C, Kastritis PL, Karaca E (2016). The HADDOCK2.2 web server: user-friendly integrative modeling of biomolecular complexes. J Mol Biol.

[39] Brouwer JM, Westphal D, Dewson G, Robin AY, Uren RT, Bartolo R (2014). Bak core and latch domains separate during activation, and freed core domains form symmetric homodimers. Mol Cell.

[40] Birkinshaw RW, Iyer S, Lio D, Luo CS, Brouwer JM, Miller MS (2021). Structure of detergent-activated BAK dimmers derived from the inert monomer. Mol Cell.

[41] Alsop AE, Fennell SC, Bartolo RC, Tan IK, Dewson G, Kluck RM (2015). Dissociation of Bak alpha1 helix from the core and latch domains is required for apoptosis. Nat Commun.

[42] Dai H, Peterson KL, Flatten KS, Meng WX, Venkatachalam A, Correia C (2023). A BAK subdomain that binds mitochondrial lipids selectively and releases cytochrome c. Cell Death Diff.

[43] Labi V, Erlacher M, Kiessling S, Manzl C, Frenzel A, O’Reilly L (2008). Loss of the BH3-only protein Bmf impairs B cell homeostasis and accelerates gamma irradiation-induced thymic lymphoma development. J Exp Med.

[44] Frenzel A, Labi V, Chmelewskij W, Ploner C, Geley S, Fiegl H (2010). Suppression of B-cell lymphomagenesis by the BH3-only proteins Bmf and Bad. Blood..

[45] Michalak EM, Jansen ES, Happo L, Cragg MS, Tai L, Smyth GK (2009). Puma and to a lesser extent Noxa are suppressors of Myc-induced lymphomagenesis. Cell Death Differ.

[46] Tan TT, Degenhardt K, Nelson DA, Beaudoin B, Nieves-Neira W, Bouillet P (2005). Key roles of BIM-driven apoptosis in epithelial tumors and rational chemotherapy. Cancer Cell.

[47] Ren D, Tu H-C, Kim H, Wang GX, Bean GR, Takeuchi O (2010). BID, BIM, and PUMA are essential for activation of the BAX- and BAK-dependent cell death program. Science..

[48] Du H, Wolf J, Schafer B, Moldoveanu T, Chipuk JE, Kuwana T (2011). BH3 domains other than Bim and Bid can directly activate Bax/Bak. J Biol Chem.

[49] Hockings C, Anwari K, Ninnis RL, Brouwer J, O’Hely M, Evangelista M (2015). Bid chimeras indicate that most BH3-only proteins can directly activate Bak and Bax, and show no preference for Bak versus Bax. Cell Death Dis.

[50] Weber K, Harper N, Schwabe J, Cohen GM (2013). BIM-mediated membrane insertion of BAK pore domain is an essential requirement for apoptosis. Cell Rep.

[51] Kim H, Tu H-C, Ren D, Takeuchi O, Jeffers JR (2009). Stepwise activation of BAX and BAK by tBID, BIM, and PUMA initiates mitochondrial apoptosis. Mol Cell.

[52] Moldoveanu T, Grace CR, Llambi F, Nourse A, Fitzgerald P, Gehring K (2013). BID-induced structural changes in BAK promote apoptosis. Nat Struct Mol Biol.

[53] Sarosiek KA, Fraser C, Muthalagu N, Bhola PD, Chang W, McBrayer SK (2017). Developmental regulation of mitochondrial apoptosis by c-Myc governs age- and tissue-specific sensitivity to cancer therapeutics. Cancer Cell.

[54] Sperl LE, Rührnößl F, Schiller A, Haslbeck M, Hagn F (2021). High-resolution analysis of the conformational transition of pro-apoptotic Bak at the lipid membrane. EMBO J.

[55] Oh KJ, Singh P, Lee K, Foss K, Lee S, Park M (2010). Conformational changes in BAK, a pore-forming proapoptotic Bcl-2 family member, upon membrane insertion and direct evidence for the existence of BH3-BH3 contact interface in BAK homo-oligomers. J Biol Chem.

[56] Meng XW, Lee SH, Dai H (2007). Mcl-1 as a buffer for proapoptotic Bcl-2 family members during TRAIL-induced apoptosis: a mechanistic basis for sorafenib (Bay 43-9006)-induced TRAIL sensitization. J Biol Chem.

[57] Honorato RV, Koukos PI, Jiménez-García B, Tsaregorodtsev A, Verlato M, Giachetti A (2021). Structural biology in the clouds: the WeNMR-EOSC ecosystem. Front Mol Biosci.

[58] Pearlman DA, Case DA, Caldwell JW, Ross WS, Cheatham TE, DeBolt S (1995). AMBER, a package of computer programs for applying molecular mechanics, normal mode analysis, molecular dynamics and free energy calculations to simulate the structural and energetic properties of molecules. Comput Phys Commun.

[59] Izadi S, Anandakrishnan R, Onufriev AV (2014). Building water models: a different approach. J Phys Chem Lett.

[60] Pastor RW, Brooks BR, Szabo A (1988). An analysis of the accuracy of Langevin and molecular dynamics algorithms. Mol Phys.

[61] Forester TR, Smith W (2000). SHAKE, rattle, and roll: efficient constraint algorithms for linked rigid bodies. J Comput Chem.

[62] Darden T, York D, Pedersen L (1993). Particle mesh Ewald: an *N*.log(*N*) method for Ewald sums in large systems. J Chem Phys.

