# Supplementary material for: Eltrombopag directly activates BAK and induces apoptosis
Source: Cell Death Dis. 2023 Jul 1;14(7):394. doi: 10.1038/s41419-023-05918-6 (PMC10314921; doi:10.1038/s41419-023-05918-6)
Supplement: Supplementary file 1 — Supplemental material [file 41419_2023_5918_MOESM1_ESM.pdf]

SUPPLEMENTAL INFORMATION TO

Eltrombopag directly activates BAK and induces apoptosis

Contains Supplementary Materials and Methods as well as Supplementary Figures S1-S8.

SUPPLEMENTARY MATERIALS AND METHODS

Materials

Reagents were purchased as followings： Lipids and extruder from Avanti Polar Lipids, Navitoclax (CT-A263) and S63845 (CT-S63845) from Chemitek, Q-VD-OPh (#HY-12305) from MedChemExpress, Ni^{2+}-NTA agarose (#17-5318-06) from GE Healthcare, allophycocyanin (APC)-labeled Annexin V (#550475) from BD Biosciences, fluorescein isothiocyanate (FITC)-labeled dextran 10 from Invitrogen, ^{15}N-labeled NH_4Cl (#299251) from Millipore-Sigma, and deuterium oxide (D_2O, #D113904) from Aladdin. Antibodies to the following antigens were purchased from the indicated suppliers and used with the indicated dilutions: Cytochrome c (#556433, 1:1000) from BD Biosciences and Hsp60 (#12165S, 1:1000) from Cell Signaling Technology. BID BH3 and BIM BH3 peptides were generated by solid phase synthesis at GL Biochem Ltd. (Shanghai, China).

Protein expression and purification

Plasmids encoding BAKΔTM (1-186) and BAK-15-186 in pET29a(+) have been described prevously.[1,2] The corresponding BAK mutants were generated using site-directed mutagenesis. All plasmids were subjected to automated sequencing to verify the intended alterations and confirm that no additional mutations were present. Plasmids were transformed into *E.coli* strain BL21. After plasmid bearing bacteria were grown to an optical density of 1.0, isopropyl β-d-1-thiogalactopyranoside was added to a final concentration of 1 mM and incubation was continued for 20 h with shaking at 16 °C. Bacteria were then washed and sonicated intermittently on ice in TS buffer (20 mM Tris-HCl, 500 mM NaCl, pH 7.4). His_6-tagged BAKΔTM and BAK-15–186 and

mutants were then applied to Ni^{2+}-NTA-agarose columns, which were washed with 20 volumes of TS buffer containing 40 mM imidazole, followed by elution with TS buffer containing 250 mM imidazole. Gel filtration with a Superdex 75 10/300 (GE Healthcare Life Sciences) was used to further purify the proteins.

Microscale thermophoresis assay

Recombinant BAKΔTM or BAKΔTM R156E was labeled at cysteine using the Monolith Protein Labeling Kit Red maleimide (NanoTemper Technologies) according to the instructions of the manufacturer. Briefly, 10 μM BAKΔTM or BAKΔTM R156E protein was incubated with 0.9 equivalents of dye in MST reaction buffer (100 mM KH_2PO_4, pH 7.4, 150 mM NaCl) in the dark at room temperature (22 °C) for 1 h. Unreacted dye was quenched using 5 mM DTT and removed using the buffer exchange column. Next, 110 nM labeled BAKΔTM or BAKΔTM R156E was incubated with increasing concentrations of Eltro in MST reaction buffer supplemented with 0.025% Tween 20. The mixtures were then loaded into standard glass capillaries (Monolith NT.115) and analyzed by MST using a Blue/Red LED power of 80%, and MST power of 80%, respectively. The fraction bound, standard deviation (SD) and K_D values were generated by NanoTemper software (MO.Affinity Analysis v2.2.4).

FPLC assay for BAK oligomerization

To perform the FPLC assay, purified BAKΔTM or mutants was mixed with small molecular compounds at the indicated molar ratio (BID BH3 as positive control and DMSO as negative control, respectively) in CHAPS buffer (20 mM HEPES, 150 mM NaCl, 1% (v/v) CHAPS, pH 7.4) and

incubated at 25 °C for 1 h. The mixture was then separated by FPLC using an S75 size exclusion column, while the protein was monitored at 280 nm. The proportion of BAK 15-186 oligomerization was calculated.

Cytochrome c release assay

To obtain mitochondria, WT, *Bak*$^{-/-}$, *Bax*$^{-/-}$, and *Bak*$^{-/-}$*Bax*$^{-/-}$ DKO MEFs were washed twice with PBS, incubated with hypotonic buffer (25 mM HEPES, 5 mM $MgCl_2$, 1 mM EGTA, 1 mM EDTA, pH 7.4) for 20 min on ice, and lysed with 30 strokes in a tight-fitting Dounce homogenizer. After KCl was added to 150 mM, nuclear fractions were removed by centrifugation at 2500 g for 15 min. After the postnuclear supernatant was sedimented at 8000 g for 15 min, the pellet (crude mitochondrial fraction) was then resuspended in hypotonic buffer with 150 mM KCl. Mitochondria were incubated with the indicated concentrations of Eltro or BID BH3 peptides at 25 °C for 90 min. After centrifugation (10,000 g, 15 min) and washing, supernatants and pellets were analyzed by immunoblotting for cyto C and Hsp60.

Cell lines

Jurkat cells were maintained at densities below 10^6 cells/mL in RPMI-1640 containing 10% heat-inactivated fetal bovine serum (FBS), 100 units/mL penicillin G, 100 μg/mL streptomycin, and 2 mM glutamine. *BAK*$^{-/-}$ Jurkat cells were generated as described.[2] MEF cells were maintained at densities below 10^6 cells/mL in DMEM containing 10% heat-inactivated fetal bovine serum (FBS), 100 units/mL penicillin G, 100 μg/mL streptomycin, and 2 mM glutamine. Cell lines were validated by short tandem repeat profiling and assayed for mycoplasma contamination regularly.

Analysis of Combination Index (CI)

Using CalcuSyn software (Biosoft, Cambridge, UK), data were analyzed by the median effect method under the assumption that effects of the two agents are mutually exclusive, which is equivalent to isobologram analysis.[3] According to this method, CI <1 indicates synergy, CI=1 indicates additivity, and CI >1 indicates antagonism.

Nuclear magnetic resonance (NMR) titration experiment

The assignments of ^{1}H,^{15}N-HSQC NMR signals of BAK 15-186 have been described before.[2] To perform NMR titration, small molecules were added to 0.2 mM ^{15}N-labeled BAK 15-186 to reach the indicated molar ratios, followed by recording of the ^{1}H, ^{15}N-HSQC of BAK 15-186. Solvent (DMSO) was used as the negative control. A series of ^{1}H,^{15}N-HSQC spectra were acquired at 303 K on a Bruker DMX850 spectrometer. Chemical Shift Perturbations (CSPs) were calculated through the formula $CSPs = [(\Delta\delta NH^2 + (\Delta\delta N/25)^2)/2]^{1/2}$. The threshold value was defined as the average CSP value plus one half standard deviation.

Stable cell lines

The cDNA encoding human BAK (GenBank BC004431) was cloned into pBabe-Puro. To allow expression in *BAK*$^{-/-}$ cells, a CRISPR/Cas9-resistant version of BAK (in which the targeted sequence of *BAK* was mutated to 5'- ACGTCAGCTGGCCATAATTG-3' and no amino acids were changed) was generated. The BAK R156E mutant was then generated by site-directed mutagenesis. Viruses were packaged in HEK293T cells by transfection using Lipofectamine 2000 (Invitrogen) with psPAX2, pMD2.G, and the pBabe vectors encoding WT or mutant BAD. After infection, cells

were selected with puromycin (2 μg/mL) for 7 days, cloned by limiting dilution and screened by immunoblotting for positive clones.

Statistical analysis

All experiments were repeated three times independently unless specifically indicated. The data was analyzed by GraphPad Prism 7 software. The data was shown as mean ± SD and one-way ANOVA Tukey's multiple comparison test was used to determine the levels of significance between comparison samples. $p > 0.05$ was not significant (ns). *, $p < 0.05$; **, $p < 0.01$; ***, $p < 0.001$; ****, $p < 0.0001$.

References:

1. Dai H, Smith A, Meng XW, Schneider PA, Pang YP, Kaufmann SH. Transient binding of an activator BH3 domain to the Bak BH3-binding groove initiates Bak oligomerization. J Cell Biol. 2011;19:39-48.
2. Ye K, Meng WX, Sun H, Wu B, Chen M, Pang Y-P, et al. Characterization of an alternative BAK-binding site for BH3 peptides. Nat Commun. 2020;11:3301.
3. Berenbaum MC. What is synergy? Phamacol Rev. 1989;41:93-141.

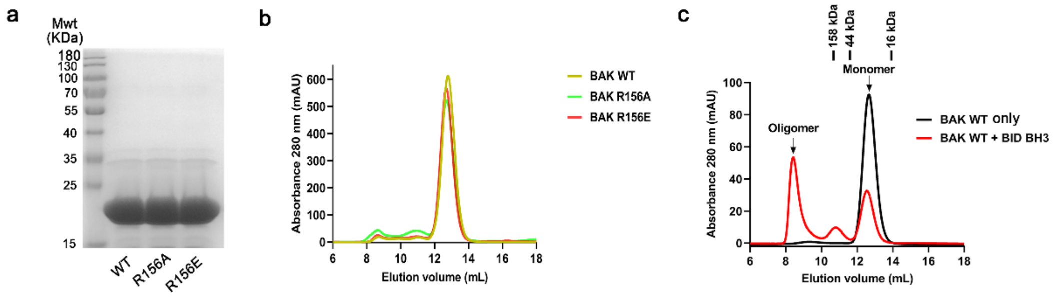

Supplementary Figure S1. Purified BAKΔTM is activated by BID BH3 peptide when analyzed using size exclusion assay

a, b Purified BAK WT, R156A, or R156E was separated by SDS-PAGE, and stained with Coomassie blue (a) or separated by FPLC on a Superdex75 column in using PBS as the mobile phase (b). **c** After BAK and BID BH3 or blank control were incubated in CHAPS buffer for 1 h at 25 °C, the mixtures were separated by FPLC on a Superdex75 size exclusion column and absorbance at 280 nm was plotted as a function of elution volume. A representative experiment out of three is shown. Labels on top indicate size markers.

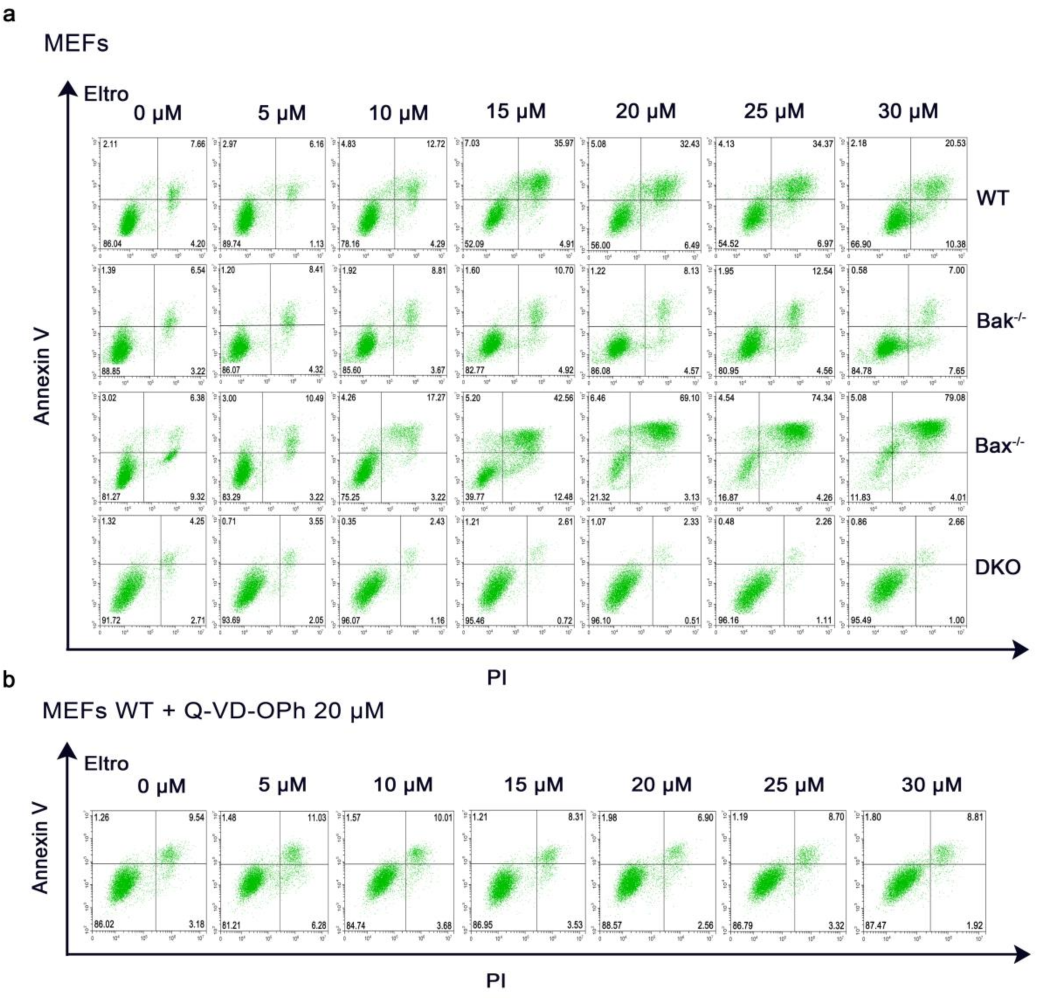

Supplementary Figure S2. Eltro induces BAK- and caspase-dependent apoptosis in MEFs.

a After WT, *Bak*$^{-/-}$, *Bax*$^{-/-}$, or *Bax*$^{-/-}$*Bak*$^{-/-}$ (DKO) MEFs cells were incubated of increasing concentrations of Eltro (0-30 μM) for 24 h, the cells were subjected to Annexin V and PI analysis by flow cytometry. **b** After WT MEFs were incubated of increasing concentrations of Eltro (0-30 μM) in the presence of Q-VD-OPh (20 μM) for 24 h, the cells were subjected to staining Annexin V and PI followed by flow cytometry.

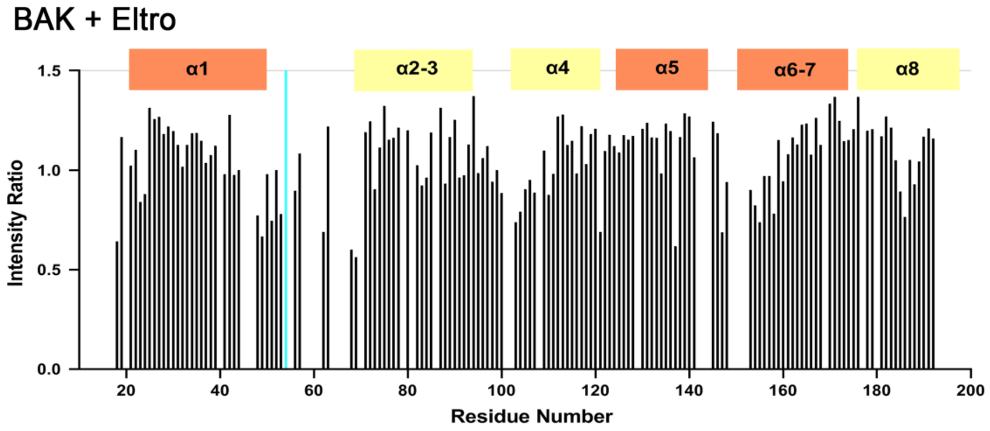

Supplementary Figure S3. Cross peak intensity changes of BAK residues from $^{1}H,^{15}N$-HSQC spectra after Eltro titration.

Intensity Ratio (left) was plotted as a function for BAK residues when $^{1}H,^{15}N$-HSQC spectra of BAK 15-186 were recorded after titration of Eltro at a ratio protein:Eltro ratio of 1:20. Residues involved in interaction were defined by peak loss (the peak intensity decreased to the level of noise), shown as the light blue bar.

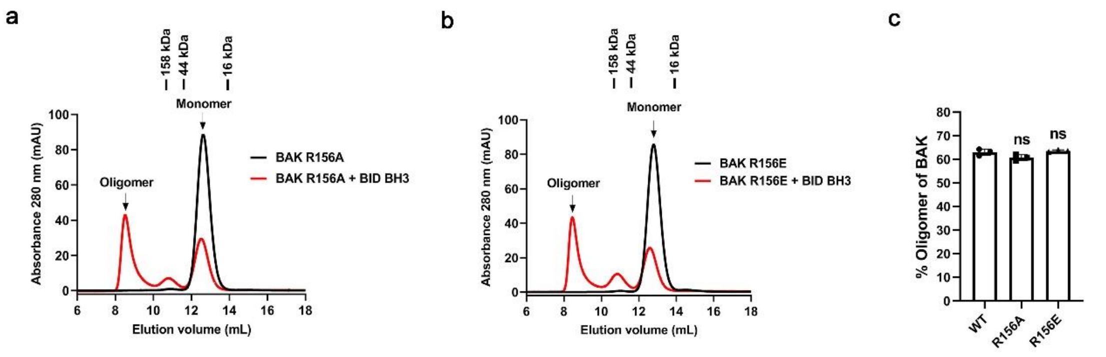

Supplementary Figure S4. BID BH3-induced BAK oligomerization is not inhibited by BAK R156A or R156E mutations.

a-c After BAKΔTM R156A or R156 (d) and BID BH3 peptide were incubated in CHAPS buffer for 1 h at 25 °C, the mixtures were separated by FPLC on a Superdex75 size exclusion column and absorbance at 280 nm was plotted as a function of elution volume. Labels on top indicate size markers. The representative data (a and b) and summarized data from three independent experiments (c) were shown.

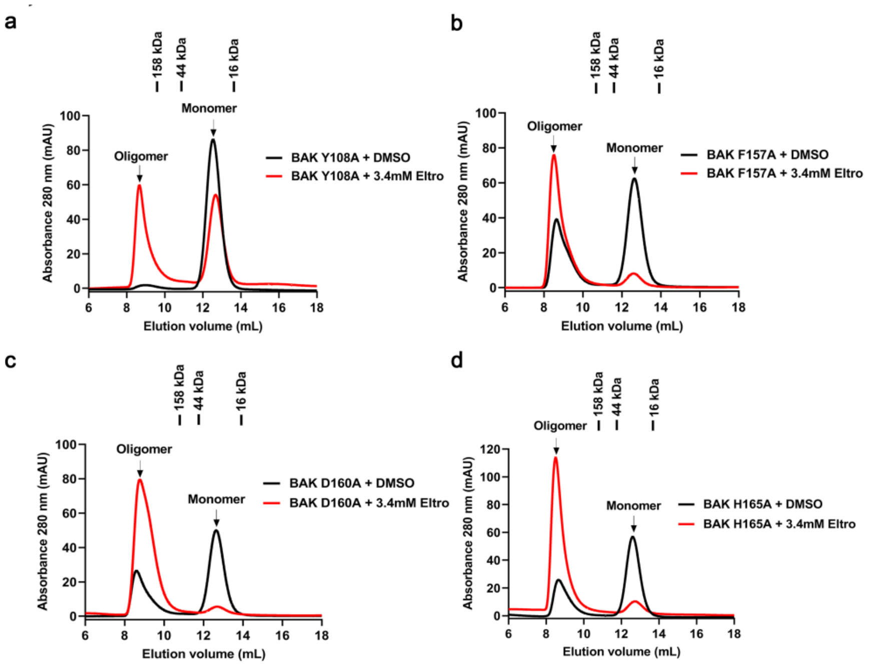

Supplementary Figure S5. Eltro-induced oligomerization of BAK mutants analyzed by size exclusion chromatography.

a-d After BAKΔTM Y108A (a), F157A (b), D160A (c) or H165A (d) and indicated concentrations of Eltro were incubated in CHAPS buffer for 1 h at 25 °C, the mixtures were separated by FPLC on a Superdex75 size exclusion column and absorbance at 280 nm was plotted as a function of elution volume. Labels on top indicate size markers.

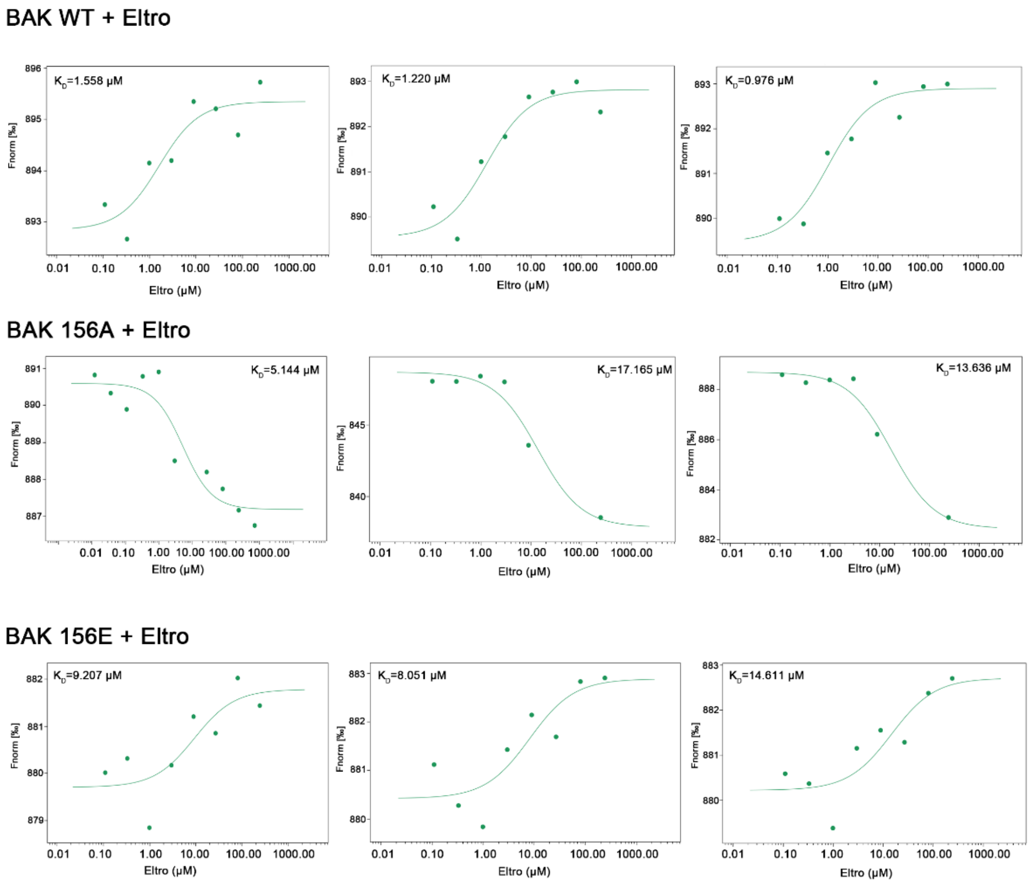

Supplementary Figure S6. BAK R156A or R156E mutation reduces BAK affinities to Eltro. After recombinant BAKΔTM WT (upper panels), BAKΔTM R156A (middle panels) or R156E (lower panels) was labeled at cysteine using Maleimide, MST analysis was performed using increasing concentrations of Eltro. Results from three independent experiments were shown.

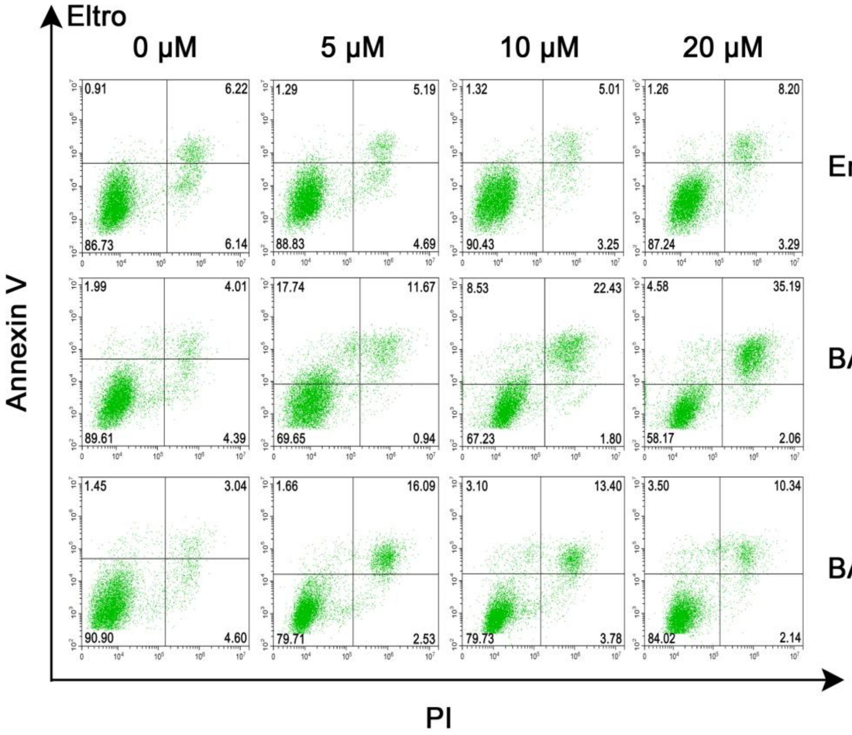

Supplementary Figure S7. Eltro induces BAK-dependent apoptosis in MEFs.

After *Bak*$^{-/-}$*Bax*$^{-/-}$ MEFs reconstituted with Empty vector, BAK WT or BAK R156E were treated with the indicated concentrations of Eltro for 24 h, cells were subjected to Annexin V/PI staining and flow cytometry. A representative experiment is shown. Summarized data from three independent experiments are shown in Figure 6i of the main text.

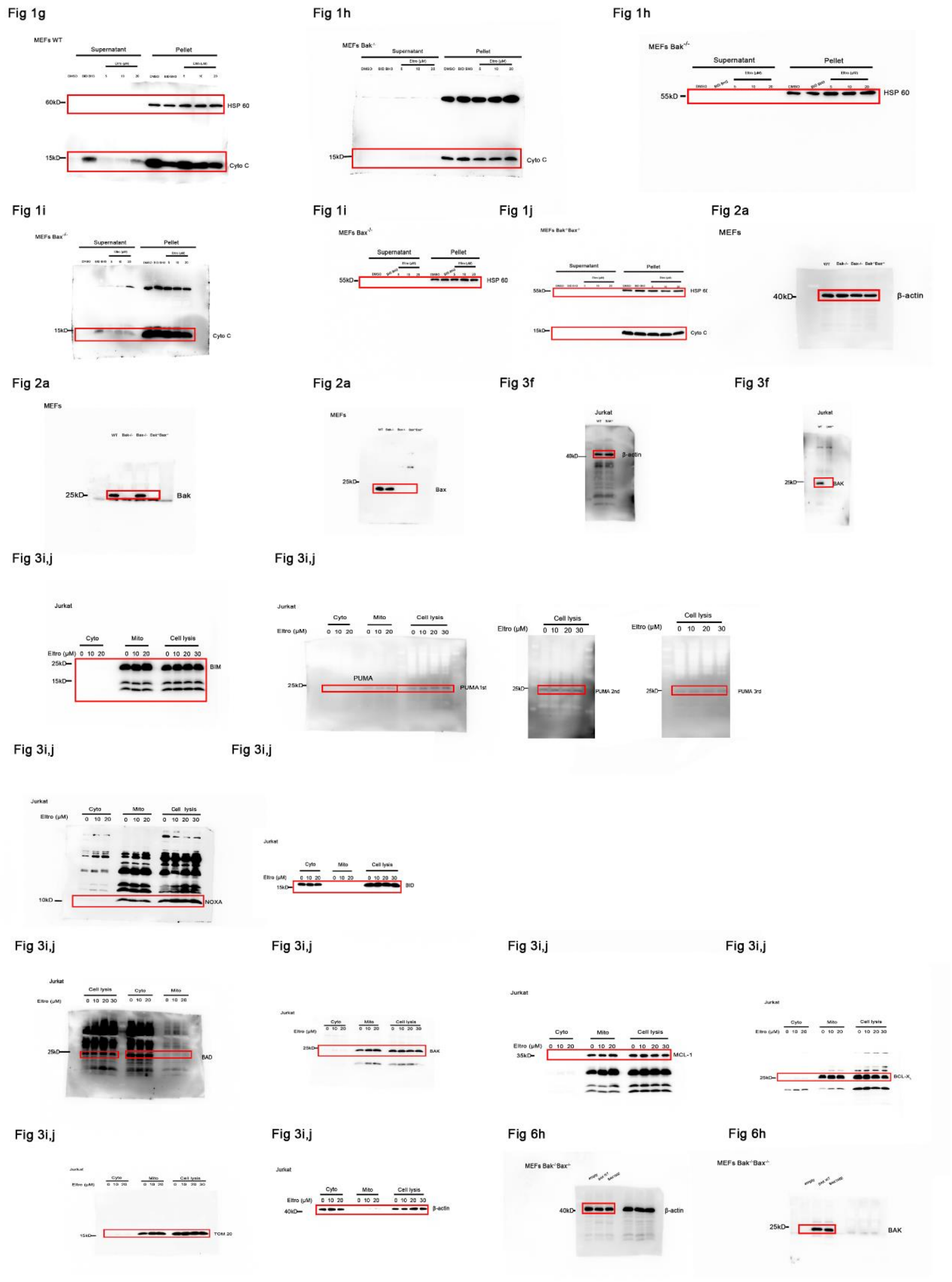

Supplementary Figure S8. Uncropped blots in this paper.
